# Effect of on-pump vs. off-pump coronary artery bypass grafting in patients with non-dialysis-dependent severe renal impairment: propensity-matched analysis from the UK registry dataset

**DOI:** 10.3389/fcvm.2024.1341123

**Published:** 2024-02-13

**Authors:** Daniel P. Fudulu, Amerikos Argyriou, Rahul Kota, Jeremy Chan, Hunaid Vohra, Massimo Caputo, Mustafa Zakkar, Gianni D. Angelini

**Affiliations:** ^1^ Department of Cardiac Surgery, Bristol Heart Institute, University of Bristol, Bristol, United Kingdom; ^2^ Bristol Medical School, University of Bristol, Bristol, United Kingdom

**Keywords:** coronary artery bypass grafting, off-pump coronary artery bypass grafting, renal dysfunction, outcomes, coronary disease

## Abstract

**Introduction:**

On-pump coronary artery bypass (ONCABG) grafting in patients with a pre-existing poor renal reserve is known to carry significant morbidity and mortality. There is limited controversial evidence on the benefit of off-pump coronary artery bypass (OPCABG) grafting in these high-risk groups of patients. We compared early clinical outcomes in propensity-matched cohorts of patients with non-dialysis-dependent pre-operative severe renal impairment undergoing OPCABG vs. ONCABG, captured in a large national registry dataset.

**Methods:**

All data for patients with a pre-operative creatinine clearance of less than 50 mL/min who underwent elective or urgent isolated OPCABG or ONCABG from 1996 to 2019 were extracted from the UK National Adult Cardiac Surgery Audit (NACSA) database. Propensity score matching was performed using 1:1 nearest neighbor matching without replacement using several baseline characteristics. We investigated the effect of ONCABG vs. OPCABG in the matched cohort using cluster-robust standard error regression.

**Results:**

We identified 8,628 patients with severe renal impairment undergoing isolated CABG, of whom 1,142 (13.23%) underwent OPCABG during the study period. We compared 1,141 propensity-matched pairs of patients undergoing OPCABG vs. ONCABG. The median age of the matched population was 78 years in both groups, with no significant imbalance post-matching in the rest of the variables. There was no difference between OPCABG and ONCABG in in-hospital mortality rates, post-operative dialysis, and stroke rates. However, the return to theatre for bleeding or tamponade was higher in ONCABG vs. OPCABG (*P* > 0.02); however, OPCABG reduced the total length of stay in the hospital by 1 day (*P* = 0.008). After double adjustment in the matched population using cluster-robust standard regression, ONCABG did not increase mortality compared to OPCABG (OR, 1.05, *P* = 0.78), postoperative stroke (OR, 1.7, *P* = 0.12), and dialysis (OR, 0.7, *P* = 0.09); however, ONCABG was associated with an increased risk of bleeding (OR, 1.53, *P* = 0.03).

**Discussion:**

In this propensity analysis of a large national registry dataset, we found no difference in early mortality and stroke in patients with pre-operative severe renal impairment undergoing OPCABG or ONCABG surgery; however, ONCABG was associated with an increased risk of return to theatre for bleeding and an increased length of hospital stay.

## Introduction

Renal dysfunction following cardiac surgery using cardiopulmonary bypass (CPB) complicates approximately 1%–5% of cases and is associated with increased mortality and morbidity (1). Its multifactorial pathogenesis includes a systemic inflammatory response or renal hypoperfusion secondary to extracorporeal circulation (1). There is evidence that off-pump coronary artery bypass (OPCABG) grafting reduces acute post-operative kidney injury compared to on-pump coronary artery bypass (ONCABG) grafting in the general cardiac surgical population (2, 3), but long-term preservation of renal function seems to remain unchanged compared to ONCABG (3). Patients with pre-operative renal dysfunction are underrepresented in large OPCABG vs. ONCABG trials; therefore, it has been suggested that avoiding CPB can mitigate some detrimental effects on renal function, ultimately translating into better inpatient outcomes (4). Small randomised controlled trial evidence suggests reduced acute chronic kidney injury using OPCABG (5). Retrospective STS registry evidence further suggests reduced in-hospital mortality and the need for renal replacement postoperatively in patients with severe non-dialysis-dependent renal impairment (6). Given the lack of studies in this area, we aimed to assess inpatient hospital outcomes of patients with pre-operative severe renal impairment (creatinine clearance of less than 50 mL/min) by comparing propensity-matched cohorts of patients undergoing OPCABG vs. ONCABG captured in a large UK registry dataset reflective of real-world practice.

## Methods

### Study design and setting

We retrospectively analysed collected data from the National Adult Cardiac Surgery Audit (NACSA), obtained from the National Institute of Cardiovascular Outcomes Research’s (NICOR) central cardiac database. The definitions of the database variables used for this study can be found at https://www.nicor.org.uk/national-cardiac-audit-programme/datasets/ in the NACSA Dataset v5.2. The NACSA registry prospectively collects demographic and pre-, peri-, and post-operative clinical data for all significant adult cardiac surgery procedures performed in the United Kingdom. Its central role is to benchmark surgical practice. The flow of the data from data input to analysis has been previously described (7). The data are entered locally and validated at the unit level by database managers before being uploaded through a web portal to NICOR.

Further validation is performed according to logical rules, and missing data reports are generated for primary variables (e.g., EuroSCORE risk factors, patient identifiers, and outcome data). The data are then forwarded to an academic healthcare informatics department for data cleaning. Duplicate records are removed, transcriptional discrepancies are re-coded, and clinical and temporal conflicts are resolved. Missing data are determined during the validation stages of the data transfer from individual centres. Missing and conflicting data for in-hospital mortality status are backfilled and validated via record linkage to the Office for National Statistics (ONS) census database. For the current study, missingness in the outcome variables in the matched population is as follows: mortality 1.3%, need for post-op dialysis 6.9%, post-operative CVA 9.2%, and length of hospital stay 0.3%. Missing data were handled by exclusion. The Health Research Authority (HRA) and Health and Care Research Wales (HCRW) approved the study in 2020 (IRAS project ID 257758), and a waiver for patients' consent was obtained. This study was conducted in accordance with the Declaration of Helsinki.

### Patients

**Figure 1:**
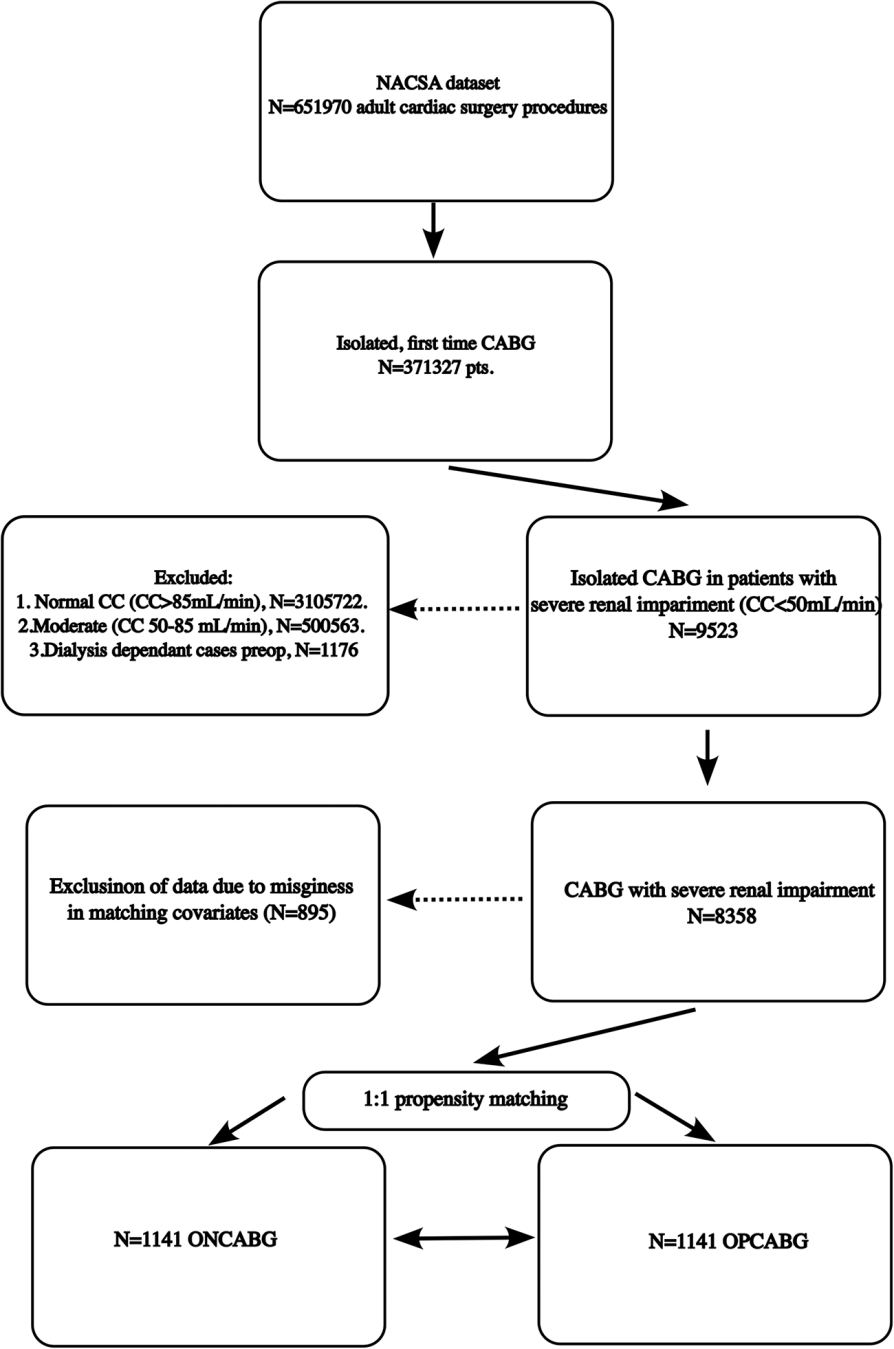
Study scheme.

We included patients who had isolated CABG and had non-dialysis-dependent severe renal impairment pre-operatively defined as a creatinine clearance of less than 50 mL/min calculated using the Cockcroft–Gault equation (8) between 18 August 1998 and 31 March 2019. Figure 1 depicts the subsetting of the patient groups included in the analysis. We excluded re-do CABG and patients with end-stage renal failure on dialysis since this is a highly comorbid patient population that is the focus of another analysis. We also excluded patients with moderate renal impairment (creatinine clearance of less than 50 mL/min) since the previous evidence suggested the most pronounced benefit of OPCABG in this spectrum of patients.

### Outcomes

The primary outcome was in-hospital mortality. Secondary outcomes included in-hospital transient ischaemic stroke (TIA), cerebrovascular accident (CVA), post-operative renal dialysis, return to theatre for bleeding or cardiac tamponade, and length of hospital stay.

### Statistical methods

Categorical variables were summarised as counts and percentages and compared using Pearson's *χ*^2^ test or Fisher's exact test. A Shapiro–Wilk test was used to assess the normality of the distribution of continuous data. Our continuous data were non-normally distributed, were summarised as a median with an interquartile range (IQR), and analysed using the Wilcoxon rank-sum test. The *P-*values were adjusted for multiple tests using the Bonferroni method.

Propensity score matching was performed using 1:1 nearest neighbor matching without replacement using the following baseline characteristics from the NACSA dataset: age, female sex, pre-operative AF, pre-operative neurological dysfunction, hypertension, body mass index (BMI), pre-operative creatinine, recent myocardial infarction, pulmonary disease, Canadian Cardiovascular Society (CCS) angina grade (0–4), New York Heart Association Heart Classification (1–4), pulmonary HTN, diabetes (diet, oral medications, or insulin), left ventricular impairment (good, moderate, and poor), peripheral vascular disease, and the urgency of surgery (elective, urgent, emergency, and salvage surgery) (Table 1).

**Table 1:** Baseline characteristics of the OPCABG and ONCABG patients in the matched cohort.

Characteristic	OPCABG, *N* = 1,141^a^	ONCABG, *N* = 1,141^a^	*P*-value^b^^–^^d^
Matched variables			
Sex: female	401 (35%)	435 (38%)	0.2
Age (years)	78 (73, 81)	78 (72, 81)	>0.9
BMI	25.6 (23.2, 28.3)	25.6 (22.9, 28.1)	0.5
Neurological dysfunction	43 (3.8%)	47 (4.1%)	0.7
Creatinine	129 (107, 161)	127 (103, 154)	0.12
Recent MI	468 (41%)	455 (40%)	0.6
Pulmonary disease	204 (18%)	172 (15%)	0.078
CCS			0.4
0	123 (11%)	114 (10.0%)	
1	90 (7.9%)	99 (8.7%)	
2	367 (32%)	342 (30%)	
3	362 (32%)	373 (33%)	
4	199 (17%)	213 (19%)	
NYHA			0.4
1	286 (25%)	244 (21%)	
2	469 (41%)	517 (45%)	
3	317 (28%)	316 (28%)	
4	69 (6.0%)	64 (5.6%)	
Pulmonary HTN	8 (0.7%)	4 (0.4%)	0.4
Diabetes			0.3
Diet controlled	47 (4.1%)	56 (4.9%)	
Oral drugs	224 (20%)	255 (22%)	
Insulin	130 (11%)	146 (13%)	
LV function			0.074
Very poor (EF ≤ 20%)	4 (0.4%)	7 (0.6%)	
Poor (EF 20%–30%)	32 (2.8%)	49 (4.3%)	
Moderate (EF 31–50%)	189 (17%)	202 (18%)	
Good (EF > 50%)	916 (80%)	883 (77%)	
Peripheral vascular disease	274 (24%)	254 (22%)	0.3
Emergency surgery	582 (51%)	590 (52%)	0.4
Urgent surgery	26 (2.3%)	34 (3.0%)	0.8
Unmatched variables			
EuroScore II	4.0 (2.8, 6.1)	4.1 (2.8, 6.2)	>0.9
Aortic cross-clamp time (min)	-	50 (38, 65)	
One graft	140 (12%)	19 (1.7%)	<0.001
Two grafts	140 (12%)	19 (1.7%)	<0.001
Three grafts	447 (40%)	619 (54%)	<0.001
Four grafts	130 (12%)	230 (20%)	<0.001
Five grafts or more	48 (4.3%)	36 (3.2%)	>0.9
Missing information on number of grafts	6 (0.5%)	3 (0.3%)	>0.9
Clinical outcomes post-matching			
Mortality	42 (3.8%)	47 (4.1%)	0.7
Post-operative dialysis	92 (8.7%)	73 (6.8%)	0.1
CVA	7 (0.7%)	15 (1.4%)	0.3
TIA	6 (0.6%)	7 (0.7%)	>0.9
Return to theatre for bleeding/tamponade	44 (3.9%)	67 (5.9%)	0.025
Total length of hospital stay (days)	11 (8, 18)	12 (8, 20)	0.008

^a^ *n* (%); Median (IQR).

^b^ Pearson's *χ*^2^ test; Wilcoxon rank-sum test; Fisher's exact test.

^c^ Bonferroni correction for multiple testing.

^d^ Paired *t*-test; McNemar's *χ*^2^ test (for matched variables).

**Figure 2:**
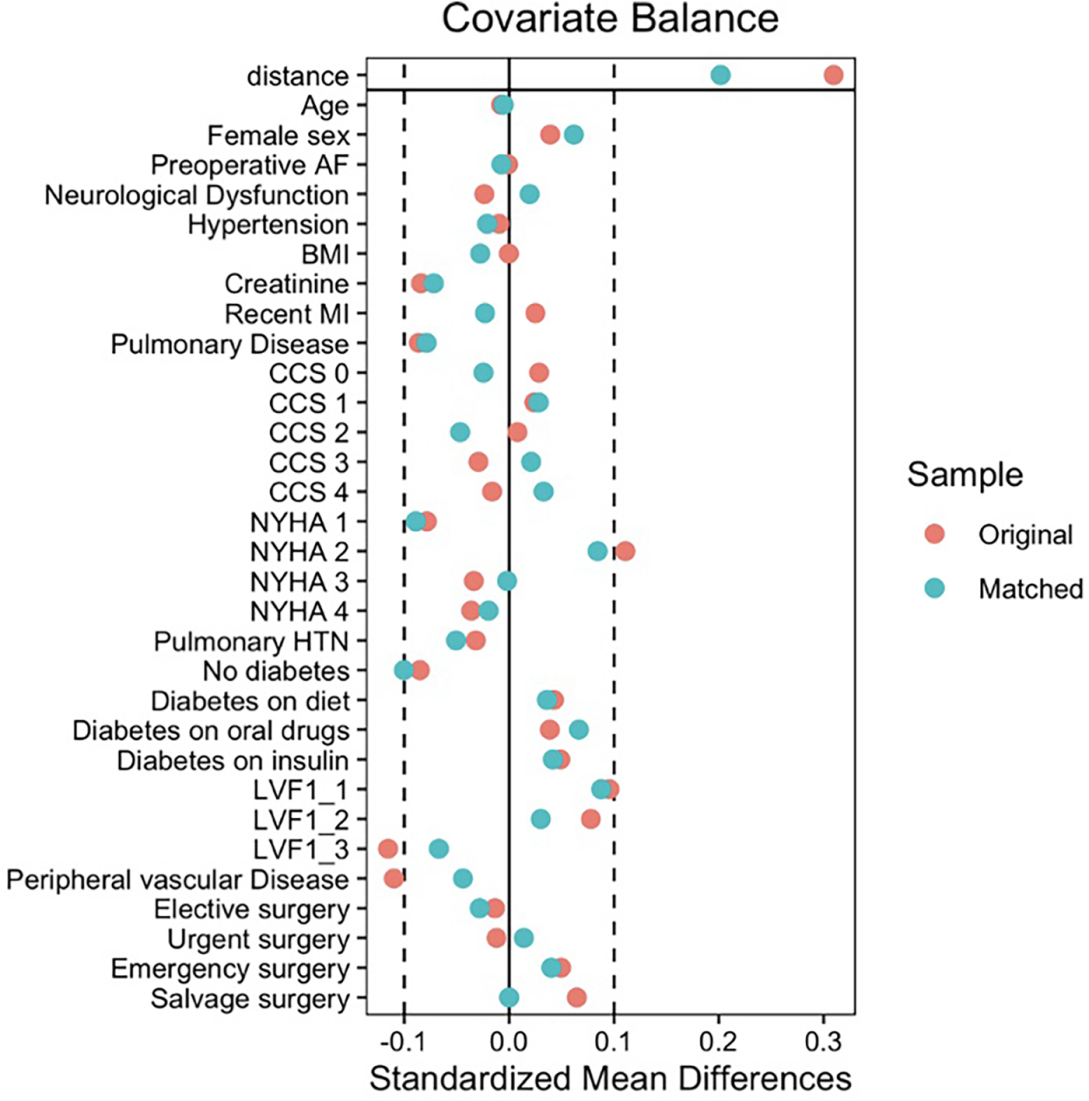
Covariate balance between unmatched and matched OPCABG and ONCABG cohorts.

After matching, all standardised mean differences (SMDs) for the covariates were checked using love plots, and the adequate balance was set to be below 0.1 (Figure 2). Matched variables were compared using a paired *t*-test for continuos data and a McNemar test for binary data (9).

To estimate the on-pump CABG effect vs. off-pump CABG and its standard error, we fitted a logistic regression model with mortality, stroke, post-op renal dialysis, and return to theatre for bleeding as binary outcomes and the treatment (on-pump CABG) and matching covariates as predictors. We included the full matching weights in the estimation. The “glm ()” function was used to fit the outcome, and the comparisons() function in the “marginal effects” package was used to perform g-computation in the matched sample to estimate the average treatment effect on patients undergoing on-pump CABG. Finally, a cluster-robust variance was used to estimate standard errors with matching stratum membership as the clustering variable (10–12). We used a composite endpoint between CVA and TIA to estimate the effect of ONCABG vs. OPCABG on stroke rates.

## Results

### Characteristics of the unmatched population

The baseline characteristics of the population we analysed are summarised in Table 2. The median age was 78 years (35% female) for the OPCABG cohort and 77 years (37% female) for the ONCABG cohort (both *P*'s > 0.9). The proportion of patients with very poor (1.3% vs. 0.3%), poor (3.8% vs. 2.8%), and moderate LV function (20% vs. 17%) was significantly higher amongst ONCABG patients (*P* = 0.01) compared to OPCABG patients. More patients with peripheral vascular disease were in the OPCABG group than in the ONCABG group (24% vs. 20%, *P* = 0.018). There were no differences in the remaining variables we used for matching (Table 1). The percentage of patients with one graft and two grafts was higher in the OPCABG group (12% vs. 1.8% and 12% vs. 1.8%, respectively, *P* < 0.001). Conversely, the percentage of patients receiving three and four grafts was higher in the ONCABG group (55% vs. 39% and 20% vs. 11%, respectively, *P* < 0.001). A very small proportion of patients underwent five grafts or more, which was higher in the OPCABG cohort than in the ONCABG cohort (4.2% vs. 2.3%, *P* = 0.004).

**Table 2:** Baseline characteristics and outcomes of OPCABG vs. ONCABG patients before matching.

Characteristic	OPCABG, *N* = 1,142^a^	ONCABG = 7,486^a^	*P*-value^b^^,^^c^
Sex: female	401 (35%)	2,770 (37%)	>0.9
Age (years)	78 (73, 81)	77 (72, 81)	>0.9
BMI	25.6 (23.2, 28.3)	25.6 (23.1, 28.4)	>0.9
Neurological dysfunction	43 (3.8%)	250 (3.3%)	>0.9
Pre-operative creatinine	129 (107, 162)	127 (104, 156)	>0.9
Recent MI	469 (41%)	3,167 (42%)	>0.9
Pulmonary disease	205 (18%)	1,114 (15%)	0.2
CCS			>0.9
0	123 (11%)	875 (12%)	
1	90 (7.9%)	640 (8.5%)	
2	368 (32%)	2,440 (33%)	
3	362 (32%)	2,272 (30%)	
4	199 (17%)	1,259 (17%)	
NYHA			0.12
1	286 (25%)	1,632 (22%)	
2	470 (41%)	3,495 (47%)	
3	317 (28%)	1,967 (26%)	
4	69 (6.0%)	392 (5.2%)	
Pulmonary HTN	8 (0.7%)	36 (0.5%)	>0.9
Diabetes			>0.9
Diet controlled	47 (4.1%)	378 (5.0%)	
Oral drugs	224 (20%)	1,587 (21%)	
Insulin	130 (11%)	975 (13%)	
LV function			0.012
Very poor (EF ≤ 20%)	4 (0.4%)	95 (1.3%)	
Poor (EF 20–30%)	32 (2.8%)	292 (3.9%)	
Moderate (EF 31–50%)	189 (17%)	1,461 (20%)	
Good (EF > 50%)	917 (80%)	5,638 (75%)	
Peripheral vascular disease	275 (24%)	1,475 (20%)	0.018
Pre-operative AF	68 (6.0%)	444 (5.9%)	>0.9
Emergency surgery	26 (2.3%)	235 (3.1%)	>0.9
Urgent surgery	582 (51%)	3,770 (50%)	>0.9
Salvage surgery	0 (0%)	31 (0.4%)	0.8
EuroScore II	4.0 (2.8, 6.1)	4.0 (2.8, 6.1)	>0.9
Aortic cross-clamp time (min)	–	49 (37, 64)	–
One graft	140 (12%)	135 (1.8%)	<0.001
Two grafts	359 (31%)	1,556 (21%)	<0.001
Three grafts	447 (39%)	4,094 (55%)	<0.001
Four grafts	130 (11%)	1,490 (20%)	<0.001
Five grafts or more	48 (4.2%)	172 (2.3%)	0.004
Missing information on number of grafts	18 (1.6%)	39 (0.5%)	0.001
Mortality	42 (3.8%)	334 (4.5%)	>0.9
Post-operative dialysis	92 (8.7%)	469 (6.6%)	0.4
CVA	7 (0.7%)	86 (1.2%)	>0.9
TIA	6 (0.6%)	62 (0.9%)	>0.9
Return to theatre for bleeding/tamponade	44 (3.9%)	393 (5.2%)	>0.9
Total length of hospital stay (days)	11 (8, 18)	12 (8, 20)	0.002

^a^ *n* (%); median (IQR).

^b^ Pearson's *χ*^2^ test; Wilcoxon rank-sum test; Fisher's exact test.

^c^ Bonferroni correction for multiple testing.

### Clinical endpoints in the unmatched population

There was no difference in mortality, post-operative dialysis, CVA, TIA, or return to theatre for bleeding or tamponade (all *P*’s > 0.005). However, the total length of hospital stay was significantly higher by 1 day in the ONCABG cohort compared to that in the OPCABG cohort (12 vs. 11 days, *P* = 0.002).

### Characteristics of the population post-matching

After matching, there was no imbalance between the baseline variables (Table 1 and Figure 1). The percentage of patients with one and two grafts remained higher in the OPCABG cohort than in the ONCABG cohort, and the percentage with three and four grafts remained higher in the ONCABG cohort (*P* < 0.001).

### Clinical outcomes post-matching

After matching, we found no difference in in-hospital mortality (4.1% vs. 3.8%, *P* = 0.7), need for post-operative dialysis (6.8% vs. 8.7%, *P* = 0.1), CVA (1.4% vs. 0.7%, *P* = 0.3), or TIA (0.7% vs. 0.6%, *P* > 0.9) between the ONCABG and OPCABG cohorts. However, return to theatre bleeding rates were higher in the ONCABG cohort than in the OPCABG cohort (5.9% vs. 3.9%, *P* = 0.008), and the length of hospital stay in the ONCABG cohort remained higher compared to that in the OPCABG cohort (12 vs. 11, *P* = 0.008).

### Effect of on-pump CABG vs. off-pump CABG on the matched population

There was no effect of ONCABG vs. OPCABG on mortality (OR, 1.05, *P* = 0.78), post-operative stroke (OR, 1.7, *P* = 0.12,) or dialysis (OR, 0.7, *P* = 0.09); however, ONCABG was associated with an increased risk of bleeding (OR, 1.53, *P* = 0.03) Table 3.

**Table 3:** Marginal odds ratios for the effect of ONCABG on mortality, post-operative stroke, post-operative dialysis, and bleeding in the matched cohort after cluster-robust logistic regression.

Predictors	Mortality			Post-operative stroke			Postoperative dialysis			Return to theatre for bleeding		
	Marginal odds ratios	95% CI	*P* Value	Marginal odds rations	CI	*P* Value	Marginal odds ratios	CI	*P* Value	Marginal odds ratios	CI	*P* Value
ONCABG	1.05	0.72–1.54	0.788	1.7	0.861–3.38	0.126	0.767	0.56–1.05	0.09	1.53	1.04–2.24	0.03

CI, confidence interval.

## Discussion

In this propensity-matched analysis of the UK dataset, we found no effect of OPCABG vs. ONCABG on patients with severe renal impairment regarding in-hospital mortality and morbidity. These results contrast with a propensity-matched analysis of 1,578 pairs of patients undergoing CABG with an eGFR of 30–59 mL/min/1.73 m^2^ by Rocha et al. (13) of the CorHealth Ontario Cardio Registry. They found OPCABG to be associated with a lower rate of in-hospital stroke and renal failure requiring dialysis and blood transfusion. In the same study, at 8-year follow-up, there was no difference in survival probability between OPCABG and ONCABG. In a review of the Society of Thoracic Surgeons (STS) registry of 742,909 non-emergent, isolated CABG cases (158,561 OPCABG), Chawla et al. (6) found OPCABG to be associated with a reduction in the composite in-hospital death or need for post-operative dialysis. Moreover, there was a greater benefit in the subset of patients with severe pre-operative renal impairment (eGFR 15–29 mL/min per 1.73 m^2^). Our results differ from the multicentre registry studies cited above for several reasons. One potential cause is the heterogeneity in the population included in the various studies and the selection criteria used to define pre-operative renal impairment. Another methodological explanation is that we used propensity matching and robust double adjustment in the matched cohorts, which further reduced residual confounding between the ONCABG and OPCABG. The only randomised study in patients with non-dialysis-dependent chronic renal impairment evaluating the OPCABG vs. ONCABG effect available to date is by Saja et al. (5) in 116 consecutive patients randomised to the ONCABG (*n* = 60) and OPCABG (*n* = 56) groups with chronic renal impairment defined by the Modification of Diet in Renal Disease equation (MDRD GFR) < ≠ 60 mL/min/1.73 m^2^. ONCABG was associated with adverse renal outcomes, requiring more blood transfusions; however, there were no differences in hospital mortality or stroke rates compared to OPCABG. In a meta-analysis by Wang et al. (4) of 201,899 patients with chronic renal impairment, including dialysis-dependent patients, pooled mostly observational studies, and one randomised trial, OPCABG reduced short-term mortality and was associated with reduced duration of ventilation and blood transfusion rates. A recent retrospective study by Phothikun et al. of 220 patients with pre-operative chronic renal impairment that compared the OPCABG with the ONCABG effect with the use of ultrafiltration found a benefit in reducing post-operative acute chronic kidney injury (14).

Our study found OPCABG to be associated with a reduced length of hospital stay, which can translate into cost savings for the NHS and improved incoming patient flow but no benefit in terms of early clinical outcomes (15). This finding is consistent with the meta-analysis by Wang et al. (4), who found OPCABG to be associated with a reduced length of hospital stay in patients with chronic renal impairment. The reduced length of hospital stay associated with OPCABG can be multifactorial and associated with the reduced morbidity observed in several studies, including reduced post-operative atrial fibrillation, which is known to be associated with prolonged hospital stay (16).

OPCABG was associated with a reduced need for post-operative transfusion in the CORONARY trial (17) and several studies in patients with pre-operative renal dysfunction (4, 5, 13). In our study, we could not assess the transfusion requirement; however, after matching and estimating the effect in the matched population, ONCABG increased the risk of return to theatre for bleeding compared to OPCABG.

### Strengths and limitations

The strength of our study is its reliance on a multicentre registry dataset that reflects real-world practice. The large size of the dataset allowed us to perform robust propensity matching and double adjustment using clustered-robust standard error regression. Nevertheless, residual confounding can remain despite the methods employed and the retrospective nature of our data, which will never equal a randomised controlled trial.

We found fewer grafts performed in the OPCABG group, consistent with well-known findings in the literature, including studies of patients with pre-operative renal dysfunction (6, 17). It would have been useful to assess the completeness of revascularisation and use these data for matching or in the prediction model. However, one of the study's limitations was high missingness and inaccuracy in pre-operative coronary disease that could not allow us to assess for incomplete revascularisation. Another limitation of our study was the lack of long-term survival data that prevented us from assessing whether the number of grafts impacts the long-term survival of patients with chronic renal dysfunction requiring CABG, which is another limitation of our study. Finally, the study spans a long period when significant improvements in cardiopulmonary bypass techniques have occurred, thus impacting any differences between the two techniques.

## Conclusion

In this propensity analysis of a large national registry dataset, we found no difference in early mortality and stroke in patients with pre-operative severe renal impairment undergoing OPCABG or ONCABG surgery; however, ONCABG was associated with an increased risk of return to theatre for bleeding and an increased length of hospital stay.

## Data Availability

The original contributions presented in the study are included in the article/Supplementary Material, further inquiries can be directed to the corresponding author.

## Supplementary material

The Supplementary rMaterial for this article can be found online at: https://www.frontiersin.org/articles/10.3389/fcvm.2024.1341123/full#supplementary-material

## References

[1] Abu-Omar Y Ratnatunga C. Cardiopulmonary bypass and renal injury. Perfusion. (2006) 21(4):209–13. 10.1191/0267659106pf870oa 16939114

[2] Garg AX Devereaux PJ Yusuf S Cuerden MS Parikh CR Coca SG Kidney function after off-pump or on-pump coronary artery bypass graft surgery: a randomized clinical trial. JAMA. (2014) 311(21):2191–8. 10.1001/jama.2014.4952 24886787

[3] Merkle J Sunny J Ehlscheid L Sabashnikov A Weber C Eghbalzadeh K Early and long-term outcomes of coronary artery bypass surgery with and without use of heart-lung machine and with special respect to renal function—a retrospective study. PLoS One. (2019) 14(10):e0223806. 10.1371/journal.pone.0223806 31600308 PMC6786630

[4] Wang Y Zhu S Gao P Zhou J Zhang Q. Off-pump versus on-pump coronary surgery in patients with chronic kidney disease: a meta-analysis. Clin Exp Nephrol. (2018) 22(1):99–109. 10.1007/s10157-017-1432-7 28634771

[5] Sajja LR Mannam G Chakravarthi RM Sompalli S Naidu SK Somaraju B Coronary artery bypass grafting with or without cardiopulmonary bypass in patients with preoperative non-dialysis dependent renal insufficiency: a randomized study. J Thorac Cardiovasc Surg. (2007) 133(2):378–88. 10.1016/j.jtcvs.2006.09.028 17258568

[6] Chawla LS Zhao Y Lough FC Schroeder E Seneff MG Brennan JM. Off-pump versus on-pump coronary artery bypass grafting outcomes stratified by preoperative renal function. J Am Soc Nephrol. (2012) 23(8):1389–97. 10.1681/ASN.2012020122 22595302 PMC3402290

[7] Hickey GL Cosgriff R Grant SW Cooper G Deanfield J Roxburgh J A technical review of the United Kingdom national adult cardiac surgery governance analysis 2008–11. Eur J Cardiothorac Surg. (2014) 45(2):225–33. 10.1093/ejcts/ezt476 24071864

[8] Cockcroft DW Gault MH. Prediction of creatinine clearance from serum creatinine. Nephron. (1976) 16(1):31–41. 10.1159/000180580 1244564

[9] Austin PC. Comparing paired vs non-paired statistical methods of analyses when making inferences about absolute risk reductions in propensity-score matched samples. Stat Med. (2011) 30(11):1292–301. 10.1002/sim.4200 21337595 PMC3110307

[10] Greifer N Stuart EA. Matching methods for confounder adjustment: an addition to the epidemiologist’s toolbox. Epidemiol Rev. (2022) 43(1):118–29. 10.1093/epirev/mxab003 34109972 PMC9005055

[11] Greifer N. Estimating effects after matching (2024). Available online at: https://cran.r-project.org/web/packages/MatchIt/vignettes/estimating-effects.html.

[12] Greifer N. MatchIt: getting started (2024). Available online at: https://cran.r-project.org/web/packages/MatchIt/vignettes/MatchIt.html.

[13] Rocha RV Yanagawa B Hussain MA Tu JV Fang J Ouzounian M Off-pump versus on-pump coronary artery bypass grafting in moderate renal failure. J Thorac Cardiovasc Surg. (2020) 159(4):1297–304.e2. 10.1016/j.jtcvs.2019.03.142 31409492

[14] Phothikun A Nawarawong W Tantraworasin A Tepsuwan T. The outcomes of ultrafiltration in on-pump versus off-pump coronary artery bypass grafting in patients with renal impairment. J Cardiothorac Surg. (2022) 17(1):219. 10.1186/s13019-022-01976-7 36045425 PMC9429667

[15] Guest JF Keating T Gould D Wigglesworth N. Modelling the annual NHS costs and outcomes attributable to healthcare-associated infections in England. BMJ Open. (2020) 10(1):e033367. 10.1136/bmjopen-2019-033367 31974088 PMC7045184

[16] Ascione R Caputo M Calori G Lloyd CT Underwood MJ Angelini GD. Predictors of atrial fibrillation after conventional and beating heart coronary surgery: a prospective, randomized study. Circulation. (2000) 102(13):1530–5. 10.1161/01.CIR.102.13.1530 11004144

[17] Lamy A Devereaux PJ Prabhakaran D Taggart DP Hu S Paolasso E Off-pump or on-pump coronary-artery bypass grafting at 30 days. N Engl J Med. (2012) 366(16):1489–97. 10.1056/NEJMoa1200388 22449296

